# The Emerging Role of Circular RNAs in Alzheimer’s Disease and Parkinson’s Disease

**DOI:** 10.3389/fnagi.2021.691512

**Published:** 2021-07-12

**Authors:** Meng Zhang, Zhigang Bian

**Affiliations:** ^1^Department of Gerontology and Geriatrics, Shengjing Hospital of China Medical University, Shenyang, China; ^2^Department of Otolaryngology Head and Neck Surgery, Shengjing Hospital of China Medical University, Shenyang, China

**Keywords:** circular RNAs, Alzheimer’s disease, Parkinson’s disease, pathophysiology, biomarker, therapy

## Abstract

Alzheimer’s disease (AD) and Parkinson’s disease (PD) are two neurodegenerative diseases (NDDs) commonly found in elderly patients that are difficult to diagnose and lack effective treatment. Currently, the available diagnostic methods for these two NDDs do not meet clinical diagnostic expectations. Circular RNAs (circRNAs) are a diverse group of endogenous non-coding RNAs (ncRNAs) found in eukaryotic cells. Emerging studies suggest that altered expression of circRNAs is involved in the pathological processes of NDDs. CircRNAs could also prove to be promising biomarkers for the early diagnosis of NDDs such as AD and PD. Growing evidence has improved our knowledge of the roles of circRNAs in NDDs, which may lead to new therapeutic approaches that target transcription for preventing neurodegeneration. In this review, we describe the formation mechanisms and functions of circRNAs as well as methods of validation. We also discuss the emerging role of circRNAs in the pathophysiology of AD and PD and their potential value as biomarkers and therapeutic targets for AD and PD in the future.

## Introduction

Neurodegenerative diseases (NDDs) are chronic progressive diseases in which neurons or the myelin sheath are damaged or die; two of the most prominent NDDs are Alzheimer’s disease (AD) and Parkinson’s disease (PD; Dugger and Dickson, 2017; Chi et al., 2018). AD is characterized by the progressive decline or loss of the normal occupational and social skills of conscious patients. The patients’ symptoms manifest as cognitive decline, significant memory loss, and impaired visual and spatial skills. Patients also experience decreased or absent orientation, calculation ability, and judgment accompanied by changes in personality, emotion, and behavior (Qiu et al., 2009; Ballard et al., 2011; Robinson et al., 2018). PD is an NDD that is common among middle-aged and elderly people and is clinically defined by tremor, myotonia, and bradykinesia (Albanese, 2003; Hayes, 2019).

A number of pathogenic pathways may be involved in the occurrence and development of NDDs. The currently recognized mechanisms of AD are amyloid β protein (Aβ) toxicity (Wang et al., 2016), microtubule-associated protein tau phosphorylation (Bakota and Brandt, 2016), cholinergic system damage (Kamkwalala and Newhouse, 2017), insulin resistance (Diehl et al., 2017), oxidative stress response (Kamat et al., 2016), blood-brain barrier (BBB) damage (Cai et al., 2018), neuroinflammatory response (Heppner et al., 2015), and deposition of α-synuclein (α-syn; Twohig and Nielsen, 2019). In PD, the degeneration of dopamine (DA) neurons in the substantia nigra (SN) of the midbrain leads to a decrease in DA in the striatum and a decrease in the activity of the SN-striatum DA transmitter system, which results in a relative increase in the cholinergic activity of the striatum (Falkenburger et al., 2016). Recent studies have shown that, in addition to the reduction of striatal dopaminergic neurotransmitters, noradrenergic, serotonergic, and acetylcholinergic transmitter systems and neuropeptides are extensively damaged in PD (Shamsuzzama and Nazir, 2017; Paredes-Rodriguez et al., 2020; Park et al., 2020). Therefore, PD is an NDD with compounded damage to multiple neurotransmitter systems.

Reliable biomarkers are critically important for the differential diagnosis of NDDs, and they also aid in early interventions and the discovery of underlying pathophysiological mechanisms of the progression of NDDs (Simrén et al., 2020). Aβ(40) levels in peripheral blood decreased significantly in patients with mild cognitive impairment (MCI) and AD (Fei et al., 2011; Janelidze et al., 2016), but the use of Aβ(40) levels in peripheral blood as a diagnostic biomarker of MCI and AD remains controversial (Lövheim et al., 2017; Yamazaki et al., 2017). The levels of total Tau (*T*-Tau) in the cerebrospinal fluid (CSF) of AD patients was two–three times that of healthy controls (Blennow, 2004). Compared with T-Tau, increased phosphorylated Tau (*P*-Tau) levels in CSF have higher specificity and could reflect pathological changes such as neurofibrillary tangles (NFTs; Buerger et al., 2006). However, due to the relatively low levels of Tau in peripheral blood, it is difficult to detect Tau in the blood of patients with MCI and AD using traditional methods (Noguchi-Shinohara et al., 2011). In PD patients, the ratio of oligomeric α-syn to total α-syn in CSF was significantly higher than that in normal controls, and thus could be used as a biomarker for diagnosis. However, the use of α-syn as a biomarker for the prognosis and cognitive impairment of PD is still debated (Reiman et al., 2011). Therefore, the hunt for biomarkers is of great importance for the diagnosis of early AD/PD in patients without clinical symptoms and could be used to monitor the course of AD/PD and the efficacy of drugs. In addition, the development of drugs that can delay or reverse AD/PD is also urgently needed.

Circular RNAs (circRNAs) are a new type of non-coding RNA (ncRNA) that are mostly circular molecules covalently connected end to end *via* 3, 5-phosphodiester bonds and do not have poly (A) structures (Kristensen et al., 2019). Most circRNAs have been found in eukaryotic cells that have certain specificity, histology, and timing (Chen and Yang, 2015). For a long time, circRNAs have been regarded as a kind of alternative splicing due to exon transcription error, resulting in a byproduct with no biological function (Hansen et al., 2013). With further study of circRNAs, researchers have found that circRNAs can act as miRNA sponges to regulate gene expression, interact with proteins, directly participate in the translation process, regulate immune responses, manage cell functions, and other mechanisms involved in the occurrence and development of various diseases, such as cancer, stroke, and type 2 diabetes mellitus (T2DM; Vijayan and Reddy, 2016; Elghoroury et al., 2018; Liang et al., 2020). Previous studies have demonstrated that miRNAs could potentially be used as biomarkers for stroke (Vijayan et al., 2018). miRNAs and lncRNAs that are differently expressed in neurological diseases may also be potential therapeutic targets, especially in AD, stroke, and T2DM (Vijayan and Reddy, 2020). Moreover, studies have shown that not only are circRNAs enriched and specifically and stably expressed in the brain tissues of humans, mice, and drosophila, but they also serve a regulatory role in synaptic remodeling and the development of the nervous system (Memczak et al., 2013; Hanan et al., 2017). Therefore, circRNAs may become functional biomarkers and therapeutic targets for a variety of diseases.

**Figure 1:**
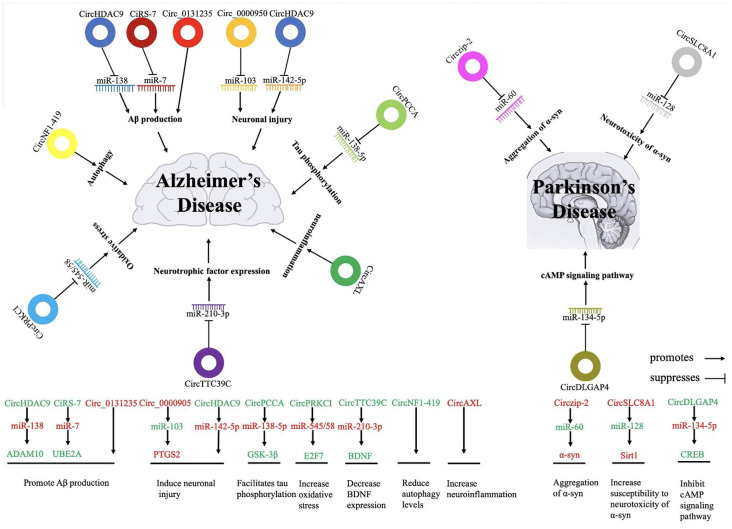
The possible roles and regulatory mechanisms and targets of circular RNAs and their impacts on the development of Alzheimer’s and Parkinson’s disease (PD). Red words indicate the upregulated non-coding RNAs and green words indicate the downregulated non-coding RNAs. circRNA, circular RNA; miR, microRNA.

In this review, we introduce the biosynthetic processes, functions, and methods of validating circRNAs, and we describe the most relevant circRNAs associated with AD/PD (Figure 1). Moreover, we will focus on the potential biomarkers involved in AD/PD diagnosis and prognosis as well as the advantages and limitations of circRNA-based treatment strategies.

## The Formation of circRNAs

CircRNAs do not have a 5′ end cap or a 3′ end poly (A) tail; rather, a covalently closed loop structure is formed by reverse splicing. These RNAs mainly originate from protein-coding genes, lncRNA, tRNA, intergenic regions, and antisense transcripts (Li Y. et al., 2015; Zheng et al., 2016). According to their constituent sequence, circRNAs can be divided into three categories: (1) circRNAs composed only of exons, known as exonic circRNAs, which are mainly located in the cytoplasm (Ma et al., 2020); (2) circRNAs composed only of introns, called intronic circRNAs, which are mainly localized in the nucleus (Zhang et al., 2013); and (3) exon-derived circRNAs that do not contain introns. However, some circRNAs retain introns between exons, called exon-intron circRNAs (EIciRNAs), and are mainly located in the nucleus (Li Z. et al., 2015).

The production mechanism of circRNAs can be divided into exon circularization and intron circularization. Two modes of exon circularization were proposed by Jeck et al. (2013): lariat-driven circularization and intron-pairing-driven circularization. Lariat-driven circularization is exon skipping that produces a lasso structure containing exons, which is then spliced within the lasso to form circRNAs. Intron-pairing-driven circularization does not rely on exon skipping; rather, the flanking introns of circularized exons can be paired with reverse complementarity through the RNA secondary structure or the abundant ALU in the introns so that the circularized exons can be spliced. This means that the position of the donor and the scissor acceptor is closer, which promotes circularization. The complementary pairing between the intron repeats flanking the exons is conducive to the formation of circRNAs. The selective pairing of inverted-repeat ALUs of different introns and the competition between them leads to a gene that can produce many different circRNAs in a process called variable circularization (Zhang et al., 2014). Furthermore, intron self- circularization can form intronic circRNAs.

## Features of circRNAs

CircRNAs are widely present and abundant in natural organisms. Many prokaryotic and eukaryotic organisms, including humans, contain numerous types of circRNAs. CircRNAs also have a high degree of tissue timing specificity. CDR1as/ciRS-7 is the best-characterized and most highly expressed of mammalian circRNAs; it is also strongly dysregulated in the hippocampal CA1 region of AD patients (Lukiw, 2013). Using *in situ* fluorescence hybridization, studies have found a high concentration of circRNAs in the nucleus or cytoplasm, such as circular ACTA2 in the nucleus (Sun et al., 2017) and circHIPK3 in cytoplasm (Xie et al., 2020). Moreover, circRNAs are strongly conserved. More than 2, 000 circRNAs in human fibroblasts can be matched to mouse genomes, and, of these, nearly one-third of the mouse genes can produce the same circRNAs (Ashwal-Fluss et al., 2014). CircRNAs are very stable in cells, with the half-life of some circRNAs in the cytoplasm exceeding 48 h; this may be because circRNAs can resist the degradation of branching enzymes and RNase R (Zhai et al., 2021).

## Biological Function of circRNAs

With the rapid development of RNA sequencing, it has been confirmed that circRNAs are not byproducts of mis-splicing but biomolecules with important regulatory functions. One of these is the miRNA sponge adsorption effect, in which miRNA binding sites on circRNAs can competitively bind to miRNA and weaken the miRNA’s regulation of the expression of its target gene (Hansen et al., 2013; Rybak-Wolf et al., 2015). Another biological function of circRNAs is to regulate parental gene expression. CircRNAs change RNA properties by base pairing, such as CDR1as and CDR1 mRNA binding complementary base pairing, to form a skeleton-like structure and increase the stability of mRNA (Hansen et al., 2013). CircRNAs can also bind to proteins and regulate their activity. Conn et al. (2015) found that circRNAs can bind to the Argonaute protein, thereby inhibiting its translation function. In addition to RNA binding protein (RBP) being involved in the formation of circRNAs, circRNAs can also form an RNA protein complex with RNA binding protein to regulate the expression of target genes in that model of circularization (Chen et al., 2015; Conn et al., 2015). CircRNA anti loci can also form pseudogenes, with at least 33 pseudogenes in the RF-WD2 locus originating from circ-RFWD2. The exon sequences of pseudogenes originating from the linear mRNA were consistent with that of the native gene, but the exon linkage sequences of pseudogenes originating from the circRNAs were opposite to those of the native gene. This indicates that circRNAs can act as retrotransposons to change genome structure and regulate gene expression (Pavlicek et al., 2006; Dong et al., 2016). CircRNA has translational potential (Lei et al., 2020). circMYBL2 has been reported to regulate FMS-like tyrosine kinase-3 (FLT3) translation by recruiting polypyrimidine tract-binding protein 1 (PTBP1) to promote FLT3-internal tandem duplication in acute myeloid leukemia progression (Sun et al., 2019).

## Identification and Validation of circRNAs

Many methods for verifying circRNAs exist. One of these is the Northern blot, which is a traditional method for gene verification. Agarose gel electrophoresis is used to design probes that will detect the existence of specific genes, but, in the case of circRNAs, the specific type of circRNA requires a different probe design from that of ordinary linear RNA. For exon-cycled circRNAs, it is recommended to cross the back splice junction site as far as possible. For intron-cycled circRNAs, probes can be designed around the intron region (Salzman, 2016; Kristensen et al., 2019). Verification of circRNAs using overexpression is another popular method for gene function determination. Overexpression of circRNAs is mainly derived from the biological formation mechanism of circRNAs. A number of studies have reported the mechanism of circRNAs circularization formation, which is currently recognized as a complementary base pairing of flanking sequences, or ALU structures, of circRNAs. Based on the characteristics of the flanking ALU sequence of circRNAs, PCR is used to amplify the target DNA sequence containing the flanking ALU sequence, digestion is performed according to the corresponding restriction enzyme sites, and the product is connected to the pEGFP-C1 vector. The vector is then transfected into the corresponding cell sample, and the transfection efficiency is detected by quantitative PCR (Darbani et al., 2016).

## circRNAs in AD

AD is one of the most common chronic NDDs, accounting for about 60% of all types of Alzheimer’s Association Report (2020) and mainly affecting the hippocampus, amygdala, temporal cortex, and frontal cortex. The pathogenesis of AD is generally believed to be related to Aβ deposition, tau protein NFTs, neuroinflammation, neurovascular impairment, and oxidative stress (Youdim, 2010; Robinson et al., 2017). CircRNAs tend to accumulate in the aging brain, and they are more abundant in mammalian brains than in other tissues (Rybak-Wolf et al., 2015; You et al., 2015). In the process of neural aging, some circRNAs accumulate significantly in neural subcellular compartments (such as synapses) compared with corresponding mRNAs; therefore, they may be considered to be a new class of aging biomarkers (Gruner et al., 2016). In addition, dysfunctional alternative splicing during aging may increase circRNA biosynthesis in the nervous system (Cortés-López et al., 2018). Specific circRNAs were highly expressed in the olfactory bulb, prefrontal cortex, hippocampus, and cerebellum (Lo et al., 2020). Synapses are one of the regions with the richest accumulation of circRNAs, and this abundance of synaptic circRNAs increases with age, suggesting that circRNAs are associated with neural development and synaptic plasticity (Gruner et al., 2016). The global accumulation of circRNA during the aging process has been discovered in different species, which indicates that circRNA may be a pathogenic factor in aging and age-related diseases, including AD (Cai et al., 2019). In fact, there are a large number of differentially expressed circRNAs in the brains of AD patients (Lo et al., 2020). Knockout/overexpression of some differentially expressed circRNAs has also been observed to alleviate AD-like pathological manifestations in cellular and animal models of AD, indicating that circRNAs are likely to be involved in the regulation of AD pathology (Table 1, Dube et al., 2019). Abnormal deposition of Aβ, abnormal autophagy, and neuronal injury are all involved in the pathological mechanism of AD. Here, we will summarize what is known about circRNAs related to the above pathological factors to better understand the role of circRNAs in the pathogenesis of AD.

**Table 1:** Studies that investigated the role of circRNAs in Alzheimer’s and Parkinson’s disease.

CircRNA	Disease/model	Changes	Function	Possible pathogenic roles in AD/PD	References
CircHDAC9	AD/*in vivo*	Decreased	miR-138/miR-142–5p sponge	Promotes Aβ production and neural cell toxicity	Lu et al. (2019)
CiRS-7	AD/*in vivo*	Decreased	miR-7 sponge	Inhibits APP and BACE1 degradation	Zhao et al. (2016); Shi et al. (2017)
Circ_0131235	AD/*in vivo*	Increased		Promote Aβ accumulation	Bigarré et al. (2021)
Circ_0000950	AD/*in vitro*	Increased	miR-103 sponge	Promotes neuron apoptosis and increases inflammatory factor levels	Yang et al. (2019)
CircTulp4	AD/*in vivo*	Decreased	Tulp4 transcription	Inhibits neuronal differentiation	Ma et al. (2021)
CircNF1-419	AD/*in vivo*	Decreased		Reduces autophagy levels	Xie et al. (2016); Diling et al. (2019)
CircPCCA	AD/*in vivo*	Decreased	miR-138–5p sponge	Facilitates tau phosphorylation	Wang et al. (2015); Li et al. (2020a)
CircAXL	AD/*in vivo*	Increased	AXL transcription	Increases neuroinflammation	Ray et al. (2017)
CircPRKCI	AD/*in vivo*	Decreased	miR-545/58 sponge	Increases oxidative stress	Cheng et al. (2019)
CircTTC39C	AD/*in vivo*	Decreased	miR-210–3p sponge	Mediates dopaminergic neuron impairment	Zhang et al. (2018)
Circzip-2	PD/*in vitro*	Decreased	miR-60 sponge	Contributes to the aggregation of α-syn	Ghosal et al. (2013)
CircSLC8A1	PD/*in vitro*	Increased	miR-128 sponge	Increases susceptibility to neurotoxicity of α-syn	Hanan et al. (2020)
CircDLGAP4	PD/*in vitro*	Decreased	miR-134-5p sponge	Inhibits cAMP signaling pathway	Feng et al. (2020)

### circRNAs Involved in Aβ Metabolism

Abnormal deposition of Aβ in the cerebral cortex is an early pathological feature of AD, which is accelerated by the abnormal processing of amyloid precursor protein (APP) and the abnormal activity of BACE1 (Alcendor, 2020). Lu et al. (2019) found that miR-138 was elevated in an age-dependent manner in APP/presenilin-1 (PS1) mice, suggesting that miR-138 could inhibit disintegrin and metalloproteinase 10 (ADAM10) expression and promote Aβ production in APP/PS1 mice. Overexpression of silent information regulator transcript 1 (Sirt1) ameliorated these miR-138-induced pathological changes *in vitro*. CircHDAC9 is a circular transcript that acts as a molecular sponge on miR-138 and is mainly localized in the cytoplasm. CircHDAC9 was decreased in the serum of AD patients, which could increase miR-138 expression and reverse Sirt1 suppression and excessive Aβ production induced by miR-138 (Lu et al., 2019).

Another mechanism that could be affected by circRNAs in AD is through UBE2A, the central effector of the ubiquitin-26 s proteasome system that coordinates the clearance of Aβ through proteolysis. UBE2A is known to be depleted in sporadic AD brains, thereby contributing to the accumulation of Aβ and the formation of senile plaque deposits. Zhao et al. (2016) suggested that the ciRS-7-miR-7-UBE2A circuit was significantly misregulated in the neocortex and hippocampal CA1 of sporadic AD patients. Deficits in ciRS-7-mediated sponging events that resulted in overexpressed miR-7 appeared to drive the significant downregulation of UBE2A expression. In addition, ciRS-7 reduced the protein levels of APP and BACE1 by promoting their degradation *via* the proteasome and lysosome. Shi et al. (2017) showed that overexpression of ciRS-7 could inhibit NF-κB translation and induce its cytoplasmic localization, thus derepressing UCHL1 and promoting BACE1 and APP degradation; this provides another regulatory mechanism of ciRS-7 in AD. Bigarré et al. (2021) found that the expression of circ_0131235 was increased in the middle temporal cortex of AD patients and may serve as a biomarker of AD pathology. They also suggested that increased circ_0131235 expression may be part of a biological mechanism to prevent damage from Aβ aggregation.

### circRNAs Involved in Neuronal Injury

Neuronal injury is a major pathological feature of AD and the most characteristic feature of NDDs. Yang et al. (2019) demonstrated that circ_0000950 downregulated miR-103 expression and upregulated prostaglandin-endoperoxide synthase 2 (PTGS2) expression in a cellular AD model that used the PC12 rat pheochromocytoma cell line and cerebral cortex neurons. Knockdown of circ_0000950 could have a therapeutic effect by enhancing neurite outgrowth, inhibiting neuron apoptosis, and lowering inflammatory factor levels *via* directly sponging miR-103. In human neuronal cells, Aβ(42) overexpression could significantly downregulate another circRNA, circHDAC9, and upregulate miR-142–5p expression. Zhang et al. (2020) suggested that increased expression of circHDAC9 could alleviate Aβ(42)-induced neuronal damage in human neuronal cells by sponging miR-142–5p, suggesting a neuroprotective role of circRNAs in alleviating neural cell toxicity in AD. Furthermore, the circTulp4 was downregulated in APP/PS1 mice, where it localized in the nucleus and interacted with RNA polymerase II and U1 small nuclear ribonucleoprotein. CircTulp4 might also regulate neuronal differentiation and influence AD development *via* promoting Tulp4 transcription (Ma et al., 2021).

### circRNAs Involved in Autophagy Processes

Accumulation of autophagosomes and abnormal autophagy are early neuropathological features of AD, which can directly affect the metabolism of Aβ (Shin et al., 2014) and accumulation of Tau, suggesting that autophagy is crucial in the pathology of AD. Enhancement of autophagy can reduce NFTs and may be a promising new therapeutic strategy for AD (Barbero-Camps et al., 2018). Studies have shown that circNF1-419 localized to the cytoplasm, and RNA pull-down experiments demonstrated that circNF1-419 could bind dynamin 1 and adaptor-related protein complex 2 subunit beta 1 protein in senescence- accelerated mice P8 (SAMP8; Xie et al., 2016). A study by Chen et al. (2015) demonstrated that overexpression of circNF1-419 in astrocytes of AD mouse models not only enhanced autophagy levels but also inhibited proteins Tau, P-Tau, Aβ(1–42), and APOE and reduced inflammatory regulators. Their results indicated that circNF1-419 could delay AD progress and defer senility, highlighting that therapeutically enhancing autophagy might be an effective strategy for AD (Diling et al., 2019). However, studies on the regulatory mechanism between circRNAs, autophagy, and AD are still scarce. More results are needed to clarify how circRNAs participate in autophagy to affect the occurrence and development of AD and provide new ideas for the diagnosis and treatment of AD.

### circRNAs Involved in Tau Phosphorylation

Tau is a cytoskeletal protein that stabilizes microtubules and is highly expressed in neurons. During AD, tau protein is hyperphosphorylated and aggregates to form NFTs (Idda et al., 2018). It has been suggested that circPCCA was decreased in the CSF of AD patients, and Li et al. (2020a) speculated that circPCCA might competitively bind to miR-138–5p to inhibit its induction of glycogen synthase kinase-3β activation and facilitate tau phosphorylation; therefore, circPCCA’s low expression was involved in exacerbating AD severity (Wang et al., 2015). Dynamin-1 is thought to provide a source of membranes for autophagosomes by pinching off vesicles from the plasma membrane (20959619).

circNF1-419 is a direct effector of Dynamin-1 binding, which is followed by Dynamin-1 mediated autophagy to eliminate AD marker proteins Tau, *p*-Tau 31860870.

### Other circRNA-Based Molecular Links in AD

CircRNAs may also be involved in AD through other pathological mechanisms. The first mechanism could be exacerbating neuroinflammation: circAXL may inhibit the transcription of its parental gene AXL, which is essential for maintaining axon integrity and inhibiting neuroinflammation, and facilitate higher susceptibility to AD (Ray et al., 2017). Another mechanism could be the promotion of oxidative stress in neurons: the expression of circPRKCI was decreased in an oxidative stress model in human primary neurons. Overexpression of circPRKCI significantly reduced the intracellular levels of miR-545/58 and protected cells from H_2_O_2_-induced apoptosis due to the circPRKCI-miR-545/589-E2F7 axis (Cheng et al., 2019). A third mechanism could be the modulation of neurotrophic factor expression: circTTC39C may sponge miR-210–3p. miR-210–3p regulates dopaminergic neuron impairment by decreasing brain-derived neurotrophic factor (BDNF), thereby protecting neurons and reducing AD risk (Zhang et al., 2018).

## circRNAs in PD

PD is another common NDD, in which DA neuron degeneration in the SN of the midbrain leads to a DA/acetylcholine imbalance. Static tremors, muscular rigidity, bradykinesia, postural abnormalities, cognitive decline, and mood disorders are the main clinical features of this disease. The pathogenesis of PD has yet to be fully understood, but current studies have shown that PD pathology may be due to mitochondrial dysfunction, excessive oxidative stress, abnormal cell apoptosis, and dysfunction of the ubiquitin-proteasome system. PD is the most common disease in the elderly and presents with motor dysfunction. Relative to the aging process, an increase in expression and accumulation of circRNAs was observed in PD brains (Jia et al., 2020). CircRNAs with significantly higher expression in aging brains were enriched for functional annotations associated with neural signaling and pathogenic processes (Rybak-Wolf et al., 2015). In fact, some analysis of circRNAs expression profiles showed differentially expressed circRNAs in varying brain regions in a PD model. Jia et al. (2020) suggested that the circRNA_0003292-miRNA-132-Nr4a2 pathway might be involved in the pathological mechanisms of PD.

### circRNAs Involved in α-Synuclein Expression

Mainly expressed in neurons, α-syn is a soluble protein that increases and accumulates in PD and is considered to be the main component of Lewy body formation (Spillantini et al., 1997). Kumar et al. (2018) found that the expression level of zip-2 increased in PD and that silencing zip-2 could significantly reduce the expression of α-syn. Circzip-2 was biosynthesized from the zip-2 gene and was subsequently discovered to be involved in the pathological mechanism of PD. Circzip-2 expression was upregulated in PD, and the parental zip-2 gene and circzip-2 were competitively expressed (Ashwal-Fluss et al., 2014). Further interaction studies demonstrated that circzip-2 might sponge miR-60, thereby asserting an important role in various processes associated with PD. Overexpression of α-syn in SN neurons is particularly involved in the pathogenesis of PD (Kraus et al., 1989). α-syn is directly inhibited by the binding of miR-7 to the 3′UTR of its target gene RNA, which suggests that downregulation of miR-7 could contribute to the aggregation of α-syn. Ghosal et al. (2013) suggested that cirs-7 may play a role in regulating the nucleoprotein enrichment pattern of miR-7 in α-syn in PD.

### circRNAs Involved in Oxidative Stress

Oxidative stress is considered to be one of the important causes of many NDDs, including PD. Hanan et al. (2020) found the circSLC8A1 expression was unregulated in the SN of individuals with PD, and circSLC8A1 levels increased in cultured cells exposed to the oxidative stress-inducing agent paraquat. CircSLC8A1 contains seven binding sites for miR-128 and can strongly bind to the miRNA effector protein Ago2. Sirt1 counteracts the neurotoxicity of α-syn and produces neuroprotective effects on PD; it also happens to be targeted by miR-128, which itself targets several mRNAs highly relevant to neurodegenerative and aging regulators. Thus, circSLC8A1 may regulate PD-related genes, such as Sirt1, by sponging miR-128.

### circRNAs Involved in Abnormal Transcription Regulation

CircRNA may also be involved in the pathogenesis and progression of PD through other mechanisms, including by regulating transcription. CircDLGAP4 is downregulated in PD models and participates in the pathophysiology of PD, and it could regulate miR-134-5p expression *via* sponging effects (Feng et al., 2020). CAMP-response element binding protein (CREB) is a target of miR-134-5p and is an important transcriptional factor in the cAMP signaling pathway that can be activated by phosphorylation at Ser133 (You et al., 2018). Activated CREB usually exerts neural protective effects by transcriptionally activating downstream target genes such as Bcl2 apoptosis regulator (Ye et al., 2019), brain-derived neurotrophic factor (BDNF; You et al., 2018), and peroxisome proliferator-activated receptor gamma coactivator 1-α (PGC-1α; Zhao and Pu, 2019). Thus, circDLGAP4 may be involved in the pathogenesis of PD *via* modulation of the miR-134-5p/CREB pathway (Feng et al., 2020).

Currently, the number of studies on circRNAs and PD is limited, but by understanding the physiological mechanisms of circRNAs, their roles in the pathological mechanisms of PD will be increasingly understood. This will also provide a basis for circRNAs to become biomarkers for the diagnosis of PD and therapeutic strategies.

## circRNAs as Biomarkers in AD and PD

With the rapid development of RNA research technologies, such as high-throughput sequencing, the mystery of circRNAs is gradually being revealed, and their close relationship with human diseases is being recognized. Recent studies have found that circRNAs are associated with various neurological diseases (Xie et al., 2017) and that they have potential as biomarkers. Previous studies have found that circRNAs can be secreted into circulating biofluid *via* exosomes and other pathways. Memczak et al. (2015) also reported that a large number of circRNAs were detected in human tissue and blood, which demonstrated the possibility of using humoral circRNAs as biomarkers for disease diagnosis. Studies on the application of circRNA detection in the diagnosis and prognosis of AD and PD are becoming hot topics in the field of circRNAs. Next, we will review the studies of circRNAs in the diagnosis of these two diseases (Table 2).

**Table 2:** CircRNAs expression as biomarkers in Alzheimer’s and Parkinson’s disease.

Disease/Samples	Patients	Method	CircRNA Alteration	Clinical significance	References
AD/Brain tissue	83 AD and 13 Ctrl	RNA sequencing	circHOMER1↑, circCDR1AS↑; circHOMER1↓	Serve as diagnostic biomarkers, predicting AD severity and explaining neuropathological status.	Dube et al. (2019)
AD/Brain tissue	19 SAD, 9ADAD, 15 Ctrl	RNA sequencing	circKCNN2↓, circHOMER1↓	Serve as diagnostic biomarkers and evaluating AD severity.	Cervera-Carles et al. (2020)
AD/CSF	8 AD and 8 Ctrl	Microarray	circLPAR1↑, circAXL↑, circGPHN↑; circPCCA↓, circHAUS4↓, circKIF18B↓	Serve as diagnostic biomarkers, predicting AD severity and explaining AD pathology.	Li et al. (2020a)
AD/PBMCs	5 AD and 5 Ctrl	Microarray	circRNA_103366↑, circRNA_103936↑, circRNA_101618↑, circRNA_405619↑, circRNA_000843↑; circRNA_104395↓, circRNA_402904↓, circRNA_403472↓	Serve as diagnostic biomarkers of AD.	Li et al. (2020b)
AD/Blood	50 AD and 50 Ctrl	Microarray	circ_0003391↓	Serve as diagnostic biomarkers, predicting AD severity and assessing anatomical changes.	Liu et al. (2020)
PD/PBMCs	60 PD and 60 Ctrl	Microarray	circ_0000497↓, circ_0000826↓, circ_0003848↓, circ_0126525↓	Serve as diagnostic biomarkers of PD.	Ravanidis et al. (2021)

*Ctrl: Control, CSF: Cerebrospinal fluid, SAD: sporadic AD, ADAD: autosomal dominant AD, PBMCs: peripheral blood mononuclear cells. The up and down arrows indicate the increase and decrease of circRNAs*.

An RNA sequencing study of cortical tissue in AD patients revealed that upregulated circHOMER1 and circCDR1-AS were significantly correlated with dementia severity. In addition, circHOMER1 was also significantly related to Braak score and AD status (Dube et al., 2019). Another study on the differential expression of circRNA in brain specimens of sporadic AD patients showed that circKCNN2 and circHOMER1 were decreased while circDOCK1 expression seemed to be enriched. Levels of circKCNN2, circHOMER1, and circFMN1 were negatively correlated with Braak scores while circDOCK1, circMAP7, circRTN4, and circPICALM were positively correlated with Braak scores. Thus, the authors suggested these circRNAs may be promising as novel AD biomarkers (Cervera-Carles et al., 2020).

Microarrays have also been used to investigate the expression profiles of circRNAs. Based on the detection of circRNAs in the cerebrospinal fluid of 8 AD patients, Li et al. (2020a) found that circAXL, circLPAR1, and circGPHN levels were increased whereas circHAUS4, circKIF18B, circPCCA, and circTTC39C levels were decreased. In addition, circITPR3, circPCCA, circGPHN, circAXL, and cicTTC39C were found to be independent predictive factors for AD risk. The levels of circGPHN and circAXL were negatively correlated with mini-mental state examination score (MMSE), whereas circHAUS4 was positively correlated. Further exploring the relationship between circRNA expression and pathological biomarkers of AD, the authors found that circKIF18B, circHAUS4, and circPCCA were positively correlated with Aβ42 while circAXL was negatively correlated. CircAXL was also positively correlated with P-tau, and circ GPHN, circAXL, and circHAUS4 were negatively correlated with *T*-tau. In peripheral blood mononuclear cells (PBMCs) of AD patients, it was reported that circRNA_000843, circRNA_101618, circRNA_103366, circRNA_103936, and circRNA_405619 were highly expressed in AD while circRNA_104395, circRNA_402904, and circRNA_403472 were lowly expressed (Li et al., 2020b).

Because circ_0003391 was significantly downregulated in the peripheral blood of AD patients, it could help to distinguish between patients with AD compared to those with other types of dementia. Liu et al. (2020) found positive correlations between the expression of circ_0003391 and the Montreal Cognitive Assessment (MoCA) and MMSE, and a negative correlation with Clinical Dementia Rating (CDR) scores. Moreover, there was a positive correlation between circ_0003391 and hippocampus volume in AD patients. Thus, circ_0003391 might be an important potential biomarker for AD as well as a reference and starting point for AD diagnostic methodology.

Differentially expressed circRNAs in PBMCs have also been studied in PD patients and have been considered as possible biomarkers for the diagnosis of PD. Ravanidis et al. (2021) enrolled 60 PD patients and 60 healthy controls to compare circRNA expression between the two groups *via* microarray analysis, which showed that six circRNAs were obviously decreased. In order to further evaluate the diagnostic value of the differentially expressed circRNAs in PD patients, receiver operating characteristic (ROC) curves were performed, and the area under the curve (AUC) was calculated to evaluate the predictive sensitivity and specificity of PBMC circRNAs for PD diagnosis. Circ_0000497, circ_0000826, circ_0003848, and circ_0126525 were all downregulated in the PD cohort, and thus could be used as biomarkers for PD diagnosis. However, the authors also admitted that these findings require further exploration.

CircRNAs have important potential in the diagnosis and prognosis of AD and PD due to their stable properties, high expression, and ability to be secreted into circulating biofluid. However, the current research on circRNAs is still in its infancy. Because there are still big gaps in the results of different detection technologies, a better universal detection method is needed, and more large-sample control studies are needed to further verify the clinical value of circRNAs as diagnostic biomarkers.

## Prospects and Conclusion

With the development of high-throughput technology, circRNAs are gradually becoming the new focus in molecular genetics following miRNA and lncRNA. In addition to the important role that circRNAs may play in the pathological mechanisms of AD and PD, they may serve as biomarkers for the diagnosis and prognosis of these diseases and even be used in the treatment of AD.

The potential of circRNA as a biomarker for AD has been previously demonstrated in small-scale clinical studies. For example, Mo et al. (2020) suggested that circAβ-A (hsa_circ_0007556) was a promising therapeutic target in both cultured cells and brains of AD patients. Compared with traditional biomarkers for AD, circRNA has the following advantages: (i) circRNA is highly expressed in the brain—higher than in other tissues and organs—and is tissue-specific (Rybak-Wolf et al., 2015); (ii) the circular structure of circRNA is more stable than that of linear RNA and is resistant to enzyme digestion (Verduci et al., 2019); and (iii) circRNAs are transferred as exosomes from the brain to bodily fluids, thus making it easier to detect them and avoiding interference from various enzymes and humoral factors (Greene et al., 2017). Therefore, peripherally circulating circRNAs have potential as diagnostic biomarkers; however, the selection of appropriate clinical samples and the identification of brain-derived circRNAs are key to exploring potential circRNA biomarkers in NDDs.

As previously mentioned, circRNAs are closely related to many pathological processes of AD/PD. It has been confirmed that circRNA can not only affect the neuronal damage of AD/PD through miRNA sponging but also bind with RNA binding protein (RBP) to regulate the differentiation and synaptic plasticity of AD/PD-related neurons. This suggests that circRNA may be an important target for designing AD/PD drugs. However, current studies on circRNA mechanisms in AD/PD are mostly *in vitro*, and the studies on circRNA functions are limited to their miRNA sponge effects and their binding to RBP. The detailed and extensive studies on the mechanisms of action of circRNAs remain to be explored, including how to avoid post-transcriptional modification of circRNAs *in vivo*. In addition, circRNA delivery methods, such as those using viruses or lipid carriers, still lack sufficient specificity, stability, and reliability. More efforts are needed to ensure that therapeutic circRNAs are delivered to the appropriate cell types, cross the BBB if necessary, and arrive at the target at the correct therapeutic concentrations. To overcome this problem, the lateral ventricle infusion strategy has been shown to improve delivery through the BBB using the ideal carrier combined with therapeutic circRNAs (Ulbrich et al., 2009). These results suggest that researchers need to explore more detailed mechanisms of circRNAs in AD/PD at the *in vivo* level.

Studies on circRNAs in NDDs are still in their infancy, and many questions remain. For instance, most studies still lack common standards for reporting and naming circRNAs (Yu and Kuo, 2019). Considering the generality and standardization of the studies, we suggest that circRNAs should be given circBase IDs in the future and named using the host gene name prefixed with “circ.” Unfortunately, current updates of circBase are very slow. If the database could be regularly updated and false positive circRNAs continuously removed, it would be helpful to further circRNA research (Glažar et al., 2014).

Another major limitation of circRNA studies is that most publicly published RNA sequence data sets related to NDDs use poly(A) purification steps to enrich mRNA, which may reduce the possibility of identifying circRNAs that naturally lack poly(A) tails (Kristensen et al., 2018). In addition, a great number of circRNAs could be detected by microarray or RNA-seq analysis, which makes correcting multiplex detection a major problem when determining the significance of differentially expressed circRNAs. However, most studies use RT-qPCR with divergent primers flanking the back-splicing junction to validate the differential expression of circRNAs, which increases the probability of selective differences. Moreover, many circRNAs have been shown to be differentially expressed *in vivo* and *in vitro*. Since most studies are retrospective, it will be interesting to observe the performance of the best circRNA biomarker candidates in large prospective clinical trials.

In conclusion, circRNA is a new type of ncRNA molecule that plays a key role in the pathological events common to NDDs, including AD and PD. It is worth noting that many circRNAs are known to be significantly dysregulated in AD/PD. Tissue-specific or circulating circRNAs may be promising diagnostic or disease progression biomarkers for AD/PD; however, a more refined understanding of the mechanisms regarding how circRNAs change as the diseases develop and progress will be required for accurate applications of circRNAs as biomarkers.

## Author Contributions

All the coauthors of this research (MZ and ZB) have directly participated in the planning and execution of the manuscript. All authors contributed to the article and approved the submitted version.

## Conflict of Interest

The authors declare that the research was conducted in the absence of any commercial or financial relationships that could be construed as a potential conflict of interest.
